# Where's Wally: the influence of visual salience on referring expression generation

**DOI:** 10.3389/fpsyg.2013.00329

**Published:** 2013-06-18

**Authors:** Alasdair D. F. Clarke, Micha Elsner, Hannah Rohde

**Affiliations:** ^1^School of Informatics, University of Edinburgh Edinburgh, Scotland, UK; ^2^Department of Linguistics, The Ohio State University Columbus, OH, USA; ^3^Linguistics and English Language, University of Edinburgh Edinburgh, Scotland, UK

**Keywords:** referring expression generation, visual salience, visual clutter

## Abstract

Referring expression generation (REG) presents the converse problem to visual search: given a scene and a specified target, how does one generate a description which would allow somebody else to quickly and accurately locate the target?Previous work in psycholinguistics and natural language processing has failed to find an important and integrated role for vision in this task. That previous work, which relies largely on simple scenes, tends to treat vision as a pre-process for extracting feature categories that are relevant to disambiguation. However, the visual search literature suggests that some descriptions are better than others at enabling listeners to search efficiently within complex stimuli. This paper presents a study testing whether participants are sensitive to visual features that allow them to compose such “good” descriptions. Our results show that visual properties (salience, clutter, area, and distance) influence REG for targets embedded in images from the *Where's Wally?* books. Referring expressions for large targets are shorter than those for smaller targets, and expressions about targets in highly cluttered scenes use more words. We also find that participants are more likely to mention non-target landmarks that are large, salient, and in close proximity to the target. These findings identify a key role for visual salience in language production decisions and highlight the importance of scene complexity for REG.

## Introduction

Cognitive science research in the domains of vision and language faces similar challenges for modeling the way people use and integrate information. For modeling people's interpretation of visual scenes and for accounting for their linguistic descriptions of such scenes, both fields must address the ways that local cues are integrated with larger contextual cues and the ways that different tasks guide people's strategies.

Despite these seemingly interlinked problem domains, vision and language have largely been studied as separate fields. Where intersections do occur, there is evidence that the way viewers make sense of a visual scene does indeed guide the language they use to describe it – visual information influences which objects speakers identify as important enough to mention and how they characterize the relationships between those objects (Coco and Keller, 2012; Clarke et al., submitted). Likewise, language itself acts as a strong gaze cue – listeners' eye movements in psycholinguistic eye-tracking experiments reflect their real-time language comprehension (Tanenhaus et al., 1995). Existing studies at the vision ~ language interface have succeeded in incorporating complex visual stimuli or complex linguistic tasks, but rarely both, and the conclusions from that previous work have assigned a limited role to vision in language production. This paper considers the question of how the language people produce in a complex referential task is influenced by the properties of a complex visual scene. Specifically, participants in our study were asked to describe individuals in illustrated crowd scenes; we then test whether the elicited descriptions reflect the visual properties of the targets themselves and of the complex scenes in which those targets appear.

In order to generate a natural and contextually appropriate description of a target object, a speaker must identify what properties of that object are relevant *in context* and what kinds of descriptions would help a listener identify that object. Understanding what people do in such tasks provides clues for improving natural language processing (NLP) systems which generate such descriptions automatically (Viethen and Dale, 2006; Krahmer and van Deemter, 2012). This task, in which a person or NLP system builds a linguistic expression to pick out a particular object in context, fits under the interdisciplinary (psycholinguistics and NLP) domain of Referring Expression Generation (REG). In order to create an appropriate description, the viewer must gather perceptual information and then compose an expression that adheres to a set of linguistic constraints. This can be thought of as the converse problem to visual search, in which an observer is given a description of the target and then has to locate it within a visual scene.

**Figure 1:**
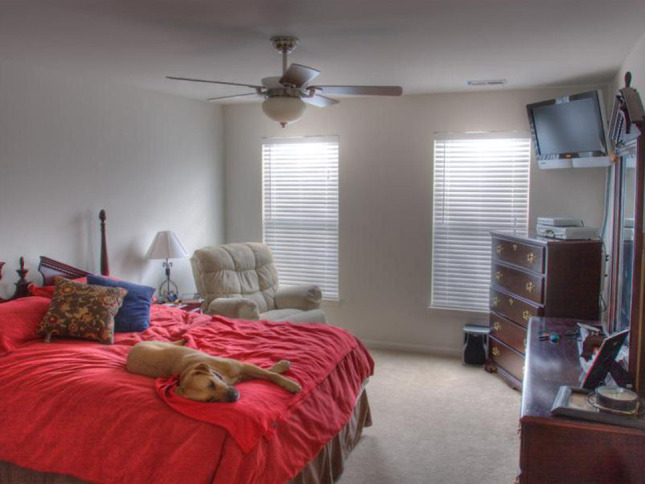
**Visually complex stimuli from a simple linguistic task (“Name as many objects as you can”; Clarke et al., submitted)**.

**Figure 2:**
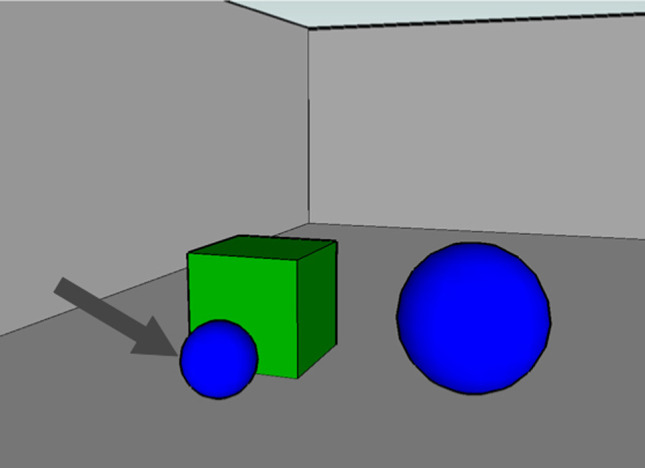
**Visually simple stimuli from a complex linguistic task (“Describe the object the arrow is pointing to”; Viethen and Dale, 2008)**.

As will be expanded on in the background sections on REG and visual perception, previous work has primarily focused on models of linguistic complexity or visual complexity but not both. Vision studies have kept the language task simple (“Describe what you see” or even just “Look at this scene”) and analyze effects from factors such as visual salience and display time (see Figure 1; and also Fei-Fei et al., 2007). On the other hand, REG studies have kept visual stimuli simple by using a small number of objects or a restricted number of feature dimensions while analyzing a more complex task (“Describe the highlighted object such that a listener could figure out which object you intended”), with the goal of evaluating the visual properties that people mention in distinguishing objects from one another (see Figure 2).

An open question is how the conclusions from such studies will scale. Given recent REG work which has concluded that the role for visual properties in complex REG tasks is small (Beun and Cremers, 1998; Viethen et al., 2011), we propose a study that uses visual scenes that approximate the detail and complexity of natural scenes. Building on evidence of differential performance in visual tasks when more abstract stimuli are used (Tatler and Melcher, 2007), our use of more complex scenes may provide a window into a very different relationship between language and vision than has previously been reported.

In our study, we investigate the role of perception in REG using images from the children's book *Where's Wally*, published in the US as *Where's Waldo.* These images are an order of magnitude more complex than the arrays of geometric objects typically used in referring expression and visual search studies, with images containing many dozens of objects and people. In our task, viewers produce a description of one highlighted target in each scene. We demonstrate an important role for visual salience (Toet, 2011) in determining which landmarks (objects other than the target) viewers choose to mention and how long a description they construct. We find that the length of viewer description reflects the size and salience of the target itself: for smaller targets, participants write longer descriptions of how to find the target (more words, more landmarks); the descriptions of the targets themselves are shorter for smaller and less salient targets (fewer target properties). We also find that the probability that an object in the scene will be chosen as a landmark reflects its own size and salience as well as its proximity to the target: large, salient landmarks are more likely to be mentioned, even at longer distances from the target.

## Referring expression generation

Early work in REG focused on the balance between brevity and descriptive adequacy – how to construct minimalist expressions that uniquely pick out the intended referent (Dale and Reiter, 1995; Krahmer and van Deemter, 2012). To describe the marked object in Figure 2, a minimalist and unambiguous expression would be one like “the small sphere.” However, it has become apparent that people do not generate minimalist expressions, instead favoring overspecification. For example, the inclusion of color terms is common even when color is not a disambiguating feature (Pechmann, 2009). To explain this pattern of overspecification, Pechmann proposed the Incremental Algorithm: speakers start producing a referring expression before they have processed sufficient information about the visual scene to know whether a particular feature of an object will disambiguate that object from others. Mitchell et al. (in press) give an extension, the Visible Objects Algorithm (VOA), which scans for potential distractor objects one-by-one and stochastically adds distinguishing characteristics to the description to rule those other objects out; the chance of adding additional information declines as the description lengthens. This makes explicit the assumption that speakers produce the expression at the same time as they visually scan the scene and that later-found distractors are less likely to matter. However Mitchell et al.'s (in press) algorithm scans objects in an arbitrary order, without reference to their visual characteristics.

The Incremental Algorithm and VOA fit within a larger psy-cholinguistics literature on audience design and common ground (Clark and Wilkes-Gibbs, 1986; Horton and Keysar, 1996; Sedivy, 2003; Brown-Schmidt and Tanenhaus, 2008). Studies in that area have documented speakers' tradeoff between listener-sensitive resource-intensive REG strategies, which take into account listener knowledge, and egocentric resource-light strategies which rely on what is visible or salient to the speaker.

Given that speakers overspecify, REG algorithms are faced with the questions of what a natural-sounding referring expression should contain and how long the expression should be. For example, do speakers focus on the target object itself or do they recruit other objects in relational descriptions in order to convey how to find the target in the larger picture? As we will show in our study, as the salience of the target object decreases, participants include more descriptions of other objects which they use as landmarks. For example, although the character Wally in each *Where's Wally* image is unique, an unambiguous description of him (“the guy with the red hat and the black-and-white striped shirt”) is insufficient to help a listener pick out the intended referent. Rather, what participants rely on are visual properties of the scene that can be referenced in the description of how to find the intended referent. The next section reviews research that has explicitly asked how visual properties influence speakers' referring expressions, and we point to the limitations with using small-scale visual scenes to answer that question.

## Influence of visual properties in REG

While the information viewers use in describing a scene must evidently depend on what they see, previous studies have generally found that visual features are only weak predictors of what people tend to say. Beun and Cremers (1998) hypothesize that salient targets will be given reduced descriptions, but due to dataset size, they do not find a significant effect. Viethen et al. (2011) show that participants describing a path through an environment delimited by groups of small colored objects (Louwerse et al., 2007) do not appear to take into account potential visual distractors when deciding what to say. This is true even in their initial references, i.e., before any referring expression has been introduced which they might be able to reuse. Viethen et al. (2011) speculate that this puzzling failure to find an effect of visual features in an explicitly visual task is due to the simple map task stimuli they used, in which perhaps “the complex mechanisms we think are required for REG more generally are simply not required” (p. 51).

Attempts to elicit relational descriptions using scenes with target objects and a set of potential landmarks have had mixed results. Viethen and Dale (2008) do find an effect of landmark salience, but in scenes with only three objects. In a more complex study of seven-object scenes (Viethen and Dale, 2011), only 13.4% of their elicited descriptions included landmarks. Moreover, they report no significant effect of landmark size (a major contributor to visual salience) on whether a landmark is mentioned.

Kelleher et al. (2005) propose a generation algorithm that incorporates visual salience and report that participants interpreted the resulting descriptions more easily when visual salience is taken into account. This result suggests an important role for visual perception, but cannot be taken as closing the issue. It is a perception study rather than a production study – it demonstrates what listeners would prefer speakers to do, rather than what they actually do. Additionally, the model of visual salience involves only two factors: an object's size and its distance to the center of the screen. Other contributing factors, like contrastive color or texture, are not measured. Nor is task relevance; the model treats the visual salience of each object in the scene as fixed, regardless of what target object is being described. In contrast, our results suggest that visual salience and relevance to a particular target interact in determining which landmarks to use in a description.

In other words, while we know visual processing is tightly integrated with perception (Sedivy et al., 1999, among others), it has been difficult to demonstrate its influence on production, at least via closely controlled studies of simple visual scenes. One possible explanation, as quoted from Viethen et al. (2011) above, is that this shows a methodological limitation of these studies. The alternative is that production involves fundamentally different visual mechanisms from perception.

This limited role for visual salience in production is the conclusion of a recent study (Gatt et al., 2012). While listeners appear to resolve referring expressions via a fast search which is sensitive to visual salience (Itti and Koch, 2000), Gatt et al. argue that speakers do not avail themselves of such cues during production. Instead, speakers perform an exhaustive scan of the objects in the scene before attempting to generate an unambiguous expression. In their study, participants identified a single target object (for example, an airplane) from a field of between 2 and 16 distractors (also airplanes, but differing in either size or color). Participants took longer to begin speaking when the number of distractors was larger, and the relationship was roughly linear. In other words, their speakers were not using an efficient visual search strategy based on salience to check whether a candidate description (“a large blue airplane”) sufficiently identifies the target.

Again, this REG result is puzzling in light of the extensive literature on perception (Eckstein, 2011; Wolfe, 2012), which shows that visual search is sensitive to visually salient features and because search and generation are in some sense converse problems – one dealing with perception, the other with production. Perceptual visual search is indeed efficient, at least for targets which contrast in certain ways with their environments (“pop-out”), and there are good models of the features which facilitate this (e.g., Guided Search; Wolfe, 1994).

We suspect that Gatt et al.'s result may reflect the types of images involved and will not necessarily generalize to more complex scenes. First, performing an exhaustive scan of a complex scene with hundreds or thousands of potential landmarks is prohibitively time-consuming. Secondly, the resulting descriptions, while guaranteed to be unambiguous, might refer to objects that listeners would nonetheless have great difficulty in finding. Lastly, the existence of completely separate visual mechanisms for perception and production (as opposed to for different types of scene) seems cognitively implausible. Although our study cannot rule out their proposal, it at least aims to establish an important role for visual salience in production as well as perception when the images involved are sufficiently complex.

## Visual search and visual salience

Models of visual salience can be thought of as modeling two related mechanisms: low-level perceptual factors that render image regions more or less apparent, and the effect that these have on visual attention. Low-level models assign scores to pixels, or regions within a scene, that reflect their visual salience: how well they stand out from their surroundings. Over the past decade many different salience models have been developed by researchers in psychology, computer vision and robotics (see Toet, 2011 for a review). Most of these models typically consider low-level features such as contrast, orientation and color, and use center-surround operations to compare the statistics of image features at a given location to the statistics in the surrounding area. These different measures have then been used by cognitive scientists who are interested in the relationship between bottom-up salience and top-down mechanisms in vision. For example, to what extent can visual salience explain the distribution of fixation locations during scene viewing (Itti and Koch, 2000; Einhauser et al., 2008)? While multiple studies have found a statistically significant effect of visual salience, these effects are often relatively weak and there are many potential confounds such as the central bias (Tatler, 2007) and correlations between objects and salient regions (Einhauser et al., 2008). In the experiments described below, we investigate whether visual salience has an effect beyond simply attracting fixations.

A related field is that of visual search. In this paradigm, participants are presented with a stimulus and asked to decide, as quickly and accurately as they can, whether a pre-specified target is present or not. Stimuli typically consist of an array of shapes (although targets embedded within photographic stimuli are also used) and the challenge to researchers is to understand how the number of search items influences the difficulty of the task. The dominant theory is Guided Search (Wolfe, 1994) in which bottom-up (visual salience) information is combined with top-down knowledge of the target's features in order to create a ranking of items which is then used to guide the deployment of visual attention. This framework succeeds in explaining how viewers search efficiently for targets that are identifiable by a single unique feature (the “pop-out” effect), while targets that are defined by a combination of feature characteristics are harder to find and typically require a serial search through the stimulus.

When more naturalistic stimuli are used in visual search studies, there is no longer a simple way to represent the number of search items in the display. Instead, visual clutter (Rosenholtz et al., 2007) has been suggested as a proxy, and the amount of clutter in a scene has been shown to correlate with the reaction times for finding a target (Henderson et al., 2009; Asher et al., 2013). Therefore we would also expect that the degree of visual clutter might influence language production, with longer descriptions being generated for targets in more cluttered scenes. We also expect clutter to influence which landmarks are selected. In particular, all objects (including potential landmarks) in cluttered scenes are expected to be harder to find, so we expect salience and area to confer more of an advantage.

The *Where's Wally* images used in this study are certainly a favorable environment to find such effects. The *Wally* series is designed as a visual search game for children. The scenes are deliberately cluttered and contain large numbers of similar-looking people as well as more and less salient objects; in some sense they represent the other extreme to the simplistic scenes used in previous work. Results on such images certainly leave open a range of intermediate visual complexity in which salience effects might be weaker and harder to detect. But we would argue that the real world looks more complex than Figure 2. For example, over the 100 photographs used in Clarke et al.'s (submitted) object naming study, subjects were asked to look at each image, then look away and list all the objects they could recall. When the lists given by 24 subjects are reconciled, they contain a median of 26 objects per image. Spain and Perona (2010) perform a similar naming experiment, but ask 5 human subjects per image to name 10 objects each, while looking at the scene. They find between 16 and 40 objects per image (median 24). The Wally images may represent the upper range of complexity in which humans must compose descriptions, but they are probably no worse than the scenes we expect people to encounter on a day-to-day basis.

## Materials and methods

### Data collection

A collection of 28 images taken from the *Where's Wally* picture books (Handford, 1987, 1988, 1993) were used as stimuli. These images depict crowded scenes and contain many cartoon people. Sixteen of these people were selected as targets by placing a 4 × 4 grid over the scene and selecting the closest person to each intersection.

Participants (*N* = 155) were recruited via Amazon Mechanical Turk, a crowd-sourced marketplace (Munro et al., 2010). Participants were asked to give their informed consent, and then they proceeded to a website that presented the Wally scenes and collected referring expressions for each target. Each participant's session consisted of two phases. First, a training phase used a search task to introduce participants to the concept of a referring expression. In training phase trials (*n* = 2), participants were given a description and asked to find the described target in a scene. The goal was to demonstrate the difference between a helpful and an unhelpful description for locating a target. The training descriptions had been collected during a pilot study, and we selected one unambiguous (helpful) description, and one ambiguous (unhelpful) description. This was done in order to show participants what makes a useful referring expression while avoiding explicit instructions (Bard et al., 2009). Following the training, participants proceeded to the main task. In each main task trial (*n* = 28), participants saw a scene with a bounding box around a target, and they were asked to write a referring expression for that target. Each participant saw each scene only once, and the 16 targets in each scene were described by 6–12 different participants.

There was no time limit for either phase of the experiment. Participants took around 5 minutes on average to complete the task and were paid 40 cents. Data from three participants was excluded: two participants completed the task twice and a third participant returned a series of one-word referring expressions. The remaining 152 participants produced a dataset of 4256 descriptions. Of that larger dataset, the results reported here use 11 of the 28 scenes; this represents the subset of the data for which we have completed annotations and consists of 1672 descriptions (152 participants × 11 trials) over 176 targets (11 scenes × 16 targets).

### Annotation

We annotated the elicited referring expressions to indicate which objects in the image were mentioned, which words in each expression referred to each object, and how the object references related to one another. Sample annotations are shown in examples (1) and (2). Words in <TARG> tags describe the target. Example (1) shows the annotated referring expression for an easy stimulus; a single landmark (the burning hut, indicated by the REL attribute) is used to localize the target. Example (2) shows the expression for a harder stimulus; two landmarks (the umbrella and ball) are introduced with the word “find” and marked with < EST> tags, and the ball is then used to localize the target. Objects in the image were labeled with bounding boxes (or for very large non-rectangular objects, bounding polygons). We did not distinguish references to geometrical parts of an object (“the left side of the track”) from references to the whole object, nor did we create separate boxes for small items that people wear or carry, or for architectural details of buildings (so “the boy in the yellow shirt” is treated as a single object). A few bounding boxes indicate groups of objects mentioned as a unit (“the three men”).

**Example (1)**

The < TARG> man < /TARG> just to the left of the < LMARK REL="TARG" OBJ="IMGID"> burning hut < /LMARK> < TARG> holding a torch and a sword < /TARG>.

**Example (2)**

Find < EST OBJ="IMGID1"> the red and white umbrella < /EST>. Then find < EST OBJ="IMGID2"> the blue and white beach ball < /EST>. Below and to the left < LMARK OBJ="IMGID2" REL="TARG"/> is < TARG> a dark skinned woman with a red bathing suit < /TARG>.

We marked the words in each expression which referred to or described each object and linked them to their corresponding bounding box. Words referring to the target were annotated with *targ* tags. When a reference to an object was used as a landmark in a relative description of another object (“the man just to the left of the *burning hut*”), we annotated it with an *lmark* ltag and indicated what object it was helping to locate.^1^ Objects mentioned without reference to another object (“find the X,” “there's an X”) were given an *est* tag (*establish*). When an expression picked out an individual without explicitly mentioning it, we created an empty phrase referring to it (so in example (2), “below and to the left” is annotated like “below and to the left *of the ball*”).

We validated our annotation scheme by independently annotating the elicited expressions for several targets in one image, then reconciling our results and updating the annotation guidelines. The authors of this paper contributed to the annotation of the referring expressions from 10 scenes, and the expressions from one additional scene were annotated by a paid annotator.

### Visual features

For each scene, we assessed the salience of our annotated landmarks and targets, and the clutter of the scene as a whole. One complicating factor is that most salience models are based on the construction of a pixel-by-pixel salience map, and therefore they do not explicitly consider an object's area to be a contributing factor to how salient it is. Indeed, many salience models tend to undervalue large objects, as they contain large homogeneous regions. However, area is a basic visual property that should be considered in any common sense definition of what makes an object more or less salient, and therefore we will also include the square root of the area of a landmark's bounding box as a visual feature along with the pixel-based salience score (below). There is a significant correlation between them, *r* = 0.38.

To compute salience scores, we use the bottom-up component of Torralba et al.'s (2006) model, which defines a salience map *S* (x, y) as:

(1) $$S(x,y)=\frac{1}{p(L(x,y)|G)}$$

where *L* and *G* are the local and global feature distributions extracted from a bank of filters (Simoncelli, 1995). The visual salience of targets and landmarks in our images is defined as being the maximum over the pixels within the relevant bounding box. Salience was predicted to guide participants' choices regarding landmark selection and description length.

We measure the distance between each proposed landmark and the search target, computed between the closest points on their respective bounding boxes. Nearby objects were predicted to be better candidates for landmark mention. Finally, we also consider the visual clutter (feature congestion) of the scene, a measure that is related to the variability of features (color, orientation, and luminance) in a local neighborhood. Full details on measuring visual clutter can be found in Rosenholtz et al. (2007).

### Data transformations

The distributions of area and distance values in the dataset are skewed to the right. This is especially true for area; the dataset contains a few very large landmarks, while objects a corresponding number of deviations below the mean would have to have negative values. To counter this, we transform the values non-linearly. We use $\sqrt{area}$ rather than area. This transformation is appropriate for several reasons (Gelman and Hill, 2007, p. 65): it makes the distribution less skewed (by visual inspection; see Figure 5; nonetheless, some outliers remain), it improves linear correlation with landmark choice, and it has a natural geometric interpretation as the width of a square bounding box.^2^ We use log(1 + *distance*) rather than distance and replace transformed values greater than 5.1 (beyond which no landmarks are ever selected, Figure 5) with 5.1; again, this makes the distribution relatively symmetric and yields an acceptable linear correlation with the output variable.

## Analysis 1: length of expressions

**Figure 3:**
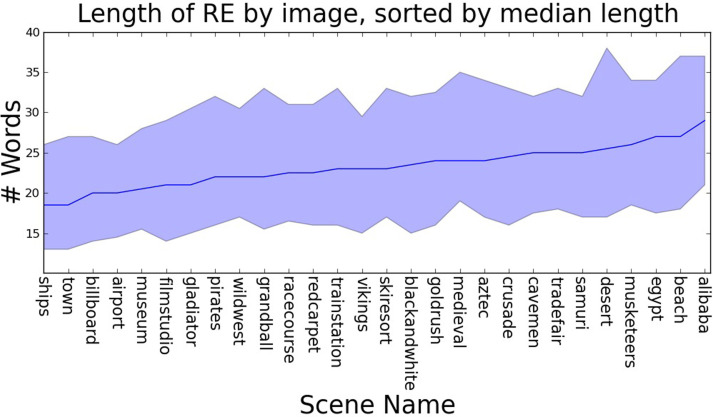
**The median length of referring expressions for targets within an image varies with scene type (computed on all 28 images)**.

**Figure 4:**
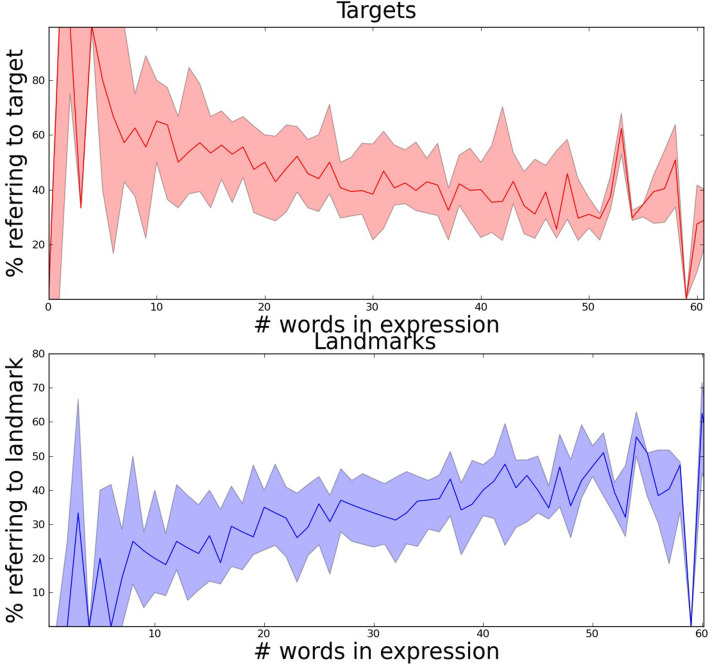
**Longer referring expressions have proportionally fewer words describing the target (top plot shows median and quartiles) and proportionally more words describing other objects (bottom plot shows median and quartiles)**.

There is a wide range in the length of the referring expressions in our dataset: between 1 and 104 words. As predicted, this variation appears to reflect the visual complexity of the scene (Figure 3): we find a correlation between the median length of referring expressions for targets in a scene and visual clutter (Spearman's rank correlation coefficient: *p* = 0.45, *p* = 0.02).^3^ We also see that the nature of referring expressions change as they get longer: short expressions typically only reference the target object, with an increasing number of landmarks being mentioned as the descriptions get longer (Figure 4).^4^

We model the length of expressions with both scene-level visual information (clutter) as well as object-level information (salience, area). We use these visual features to predict three outcomes: the total number of words in a description, the proportion of words referencing the target, and the number of landmarks mentioned. For the number of words and number of landmarks, we use linear mixed-effects models with a poisson linking function to model the count values. For the proportion of words referencing the target, we use a logistic mixed-effects model. All models contained factors for the salience and square root area of the target, the visual clutter of the scene, and interactions among those three factors.

Models were fit using the lmer function of the R package lme4 (Bates et al., 2011), with random intercepts for both participants and target and random slopes (fully crossed) for participants. These models estimate the size and direction of main effects and their interactions while simultaneously including baselines for individual participants and targets. We report the coefficient estimates, standard error, *t*-value, and MCMC-derived *p*-values (Baayen et al., 2008). All predictors were centered so that the main effects remain interpretable.

For the overall number of words in the description, there was a main effect of area and marginal effects of salience and visual clutter (Table 1): descriptions were shorter for targets with larger area (β = −0.04) and greater salience (β = −0.03) and longer for targets in scenes with high clutter scores (β = 0.03). A marginal area × salience interaction indicated that these two negative effects are not quite additive, with a slightly reduced effect when both are large (β = 0.02). None of the other interactions reached significance.

**Table 1:** **Results of mixed-effects model for predicting number of overall words in a description**.

	**β**	**SE**	***t*-value**	***p*-value**
**Area**	**−0.04**	**0.02**	**−2.29**	**<0.05**
Salience	−0.03	0.02	−1.76	0.08
Clutter	0.03	0.02	1.74	0.08
Area × sal	0.02	0.01	1.78	0.08
Area × clutter	−0.02	0.02	−0.92	0.36
Sal × clutter	0.01	0.02	0.76	0.45
Area × sal × clutter	−0.01	0.02	−0.27	0.79

*Bolding indicates main effects or interactions that reached significance.*

For the proportion of words referencing the target itself, there were main effects of area and salience (Table 2). Target descriptions were longer for those targets with larger area (β = 0.25) and greater salience (β = 0.20). There was no effect of clutter and the only interaction to reach significance was again the area × salience interaction, whereby the overall effect of these two factors is reduced when both are large (β = −0.11).

**Table 2:** **Results of mixed-effects model for predicting proportion of words referencing the target in a description**.

	**β**	**SE**	***z*-value**	***p*-value**
**Area**	**0.25**	**0.05**	**5.11**	**<0.001**
**Salience**	**0.20**	**0.05**	**4.25**	**<0.05**
Clutter	−0.02	0.04	−0.52	0.60
**Area** × **sal**	**−0.11**	**0.04**	**−2.78**	**<0.01**
Area × clutter	0.02	0.05	0.34	0.73
Sal × clutter	0.02	0.06	0.45	0.65
Area × Sal × clutter	−0.04	0.05	−0.57	0.57

*Bolding indicates main effects or interactions that reached significance.*

For the number of landmarks included in the description, there were likewise effects of area and salience (Table 3). The number of landmarks mentioned decreased for targets with larger area (β = −0.14) and greater salience (β = −0.12). Again, area and salience interact (β = 0.07). Neither clutter nor any of the other interactions reached significance.

**Table 3:** **Results of mixed-effects model for predicting number of landmarks included in a description**.

	**β**	**SE**	**z-value**	***p*-value**
**Area**	**−0.14**	**0.03**	**−4.01**	**<0.001**
**Salience**	**−0.12**	**0.04**	**−3.50**	**<0.001**
Clutter	0.00	0.03	−0.13	0.89
**Area** × **Sal**	**0.07**	**0.03**	**2.40**	**<0.05**
Area × Clutter	−0.01	0.03	−0.25	0.80
Sal × Clutter	−0.05	0.04	−1.25	0.21
Area × Sal × Clutter	−0.02	0.04	−0.56	0.58

*Bolding indicates main effects or interactions that reached significance.*

## Analysis 2: choice of landmarks

**Figure 5:**
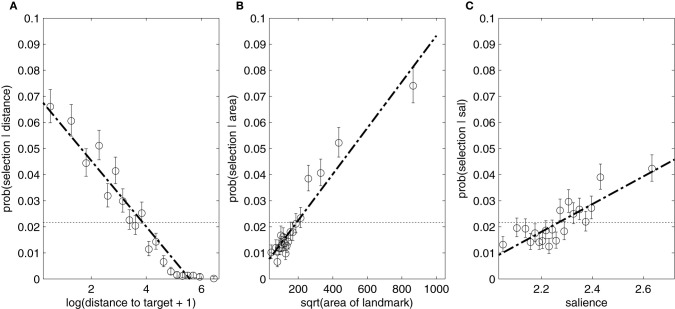
**The effect of each feature on the conditional probability of naming a given object: (A) distance, (B) area, (C) salience**. These figures were generated by dividing each feature range into 5% quantiles, and plotting the probability of mentioning a landmark from that quantile. Binomial distributions were then fitted to provide confidence intervals. The dotted line shows an estimate of the prior probability of selecting a landmark.

The effects of an individual landmark's features on the probability of that landmark being chosen in an expression are shown in Figure 5. To measure the effect of visual properties on the choice to mention a particular landmark in a referring expression, we modeled the binary outcome of mention for each landmark in each description using a mixed-effects logistic regression. The model contained factors for the salience and square root area of the landmark, the distance between the landmark and the target, the visual clutter of the scene, and interactions among those four factors. Random participant-specific and target-specific intercepts and slopes were included (slopes were not crossed, due to the number of parameters to estimate and problems with model convergence). For this model, all objects in a scene were included, meaning that the mention outcome was 0 for most landmarks relative to most targets, since only a few landmarks were near enough or large/salient enough to merit mention. The set of ‘all objects’ consisted of every object that was mentioned in at least one referring expression in the dataset.

Again, we used the lmer function of the R package lme4. We report the coefficient estimates, standard error, and p-values based on the Wald Z statistic (Agresti, 2002). All predictors were centered.

The results (Table 4) show main effects of area, distance, and crucially visual salience: a landmark is more likely to be mentioned the larger it is (area: β = 0.57) and the more salient it is (salience: β = 0.25); it is less likely to be mentioned the farther it is from the target (distance: β = −0.99). The positive effect of area was stronger in more cluttered scenes (area × clutter: β = 0.20) and at greater distances (area × distance: β = 0.21), and this interaction with distance was stronger in more cluttered scenes (area × distance × clutter: β = 0.11). Again area and salience interact, reducing their overall effect when both are large (area × sal: β = −0.22), though this is less apparent in more cluttered scenes (area × salience × clutter interaction: β = −0.19) and at greater distances (area × salience × distance interaction: β = −0.09). Finally, the 4-way interaction was significant (area × salience × distance × clutter: β = −0.05), meaning that more distant, larger salient objects are less likely to be selected in cluttered scenes.

**Table 4:** **Results of mixed-effects model for predicting whether a landmark would be included in a description**.

	**β**	**SE**	***p*-value**
**Lmark Area**	**0.57**	**0.05**	**<0.001**
**Lmark Salience**	**0.25**	**0.11**	**<0.05**
**Dist to targ**	**−0.99**	**0.05**	**<0.001**
Clutter	0.11	0.07	0.10
**Area** × **Sal**	**−0.22**	**0.04**	**<0.001**
**Area** × **Dist**	**0.21**	**0.03**	**<0.001**
**Area** × **Clutter**	**0.20**	**0.05**	**<0.001**
Sal × Dist	0.04	0.03	0.23
Sal × Clutter	−0.03	0.11	0.78
Dist × Clutter	0.05	0.05	0.31
**Area** × **Sal** × **Dist**	**−0.09**	**0.02**	**<0.001**
**Area** × **Sal** × **Clut**	**−0.19**	**0.03**	**<0.001**
**Area** × **Dist** × **Clut**	**0.11**	**0.03**	**<0.001**
Sal × Dist × Clut	0.00	0.03	0.99
**Area** × **Sal** × **Dist × Clut**	**−0.05**	**0.02**	**<0.05**

*Bolding indicates main effects or interactions that reached significance.*

## Discussion

These results demonstrate that participants' production of referring expressions is affected by their perception of visual salience and clutter. As stated above, we agree with Viethen et al. (2011) that previous studies failed to show such clear effects because their stimuli were too simple. The fact that cluttered scenes correlate with longer referring expressions overall and that the effect of landmark size on landmark selection is greater in cluttered scenes suggests that it is indeed the visual complexity of these scenes that renders an object's visual properties important. The beach scene, for instance, has hundreds of similarly sized and colored human figures which are generally poor choices as landmarks, since most of them are no easier to find than the targets. Objects like the red and white umbrella, however, “pop-out” of the scene, facilitating efficient visual search.

Of course, salience is not the only driving force behind landmark selection. Participants might select landmarks with lower computed salience for a variety of reasons. In some cases, these landmarks appear to be intended as confirmation that the right object has been found, rather than an aid in finding the object to begin with. In others, their attention might be strongly directed toward the region around the target, so that objects appear *perceptually* salient to them despite not being *visually* salient to an observer who is unaware of the target's location. Such task-based effects on gaze and attentional allocation are known from other studies (Land et al., 1999).

This raises the further question of how closely our computational salience prediction algorithm corresponds to actual human perception. Certainly it contributes something more than simple area and centrality (the model of salience implemented in Kelleher et al., 2005). We are currently performing visual search experiments in which participants are asked to find the targets and landmarks used in this study given non-linguistic instructions in the form of thumbnail images. This should help us decide how well the Torralba et al. (2006) system is predicting what participants actually see when they look at a scene. If it is doing a relatively good job, many landmarks that appear non-salient may have been selected due to task effects; otherwise, they may in fact be salient in ways unrepresented by the model.

While we have shown that salience has an effect on referring expression production, a critical question remains: do speakers choose to talk about salient objects in order to save themselves visual work, or do they perform a relatively comprehensive scan, but prefer to talk about objects that will be easier for listeners to find? In other words, is the observed effect driven by participant efficiency, or is it a case of “audience design” in which speakers try to make listeners' tasks efficient? REG models like the incremental algorithm of Pechmann (2009) would predict speaker efficiency effects, while minimal-description (Gricean) models like Dale and Reiter (1995) predict audience design.

The current study is insufficient to resolve this question. Although a negative finding (that visual salience had no effect) would have been fatal for the incremental model, the minimal-description model can incorporate visual salience (as in Kelleher et al., 2005) by modifying its utility function to prefer descriptions that are visually efficient rather than simply short. The models do make differing predictions about real-time processing, however. The incremental algorithm suggests speakers select landmarks by rapidly scanning near the target for visually salient objects. Minimalist models predict that the speaker makes a slower and more exhaustive scan to build a list of potential landmarks, then selects among them according to the utility function. The reaction time study of Gatt et al. (2012) found support for the minimalist model, but on visual stimuli of the type for which visual salience typically has little effect on REG. We conjecture that the results might be different on our stimuli, and intend to test this hypothesis in the future.

Beyond REG, our results also contribute to the ongoing debate surrounding the importance of salience in visual perception. Since the introduction of computational salience models, vision scientists have been able to test predictions from these models and compare them to the distributions of fixations obtained during eye-tracking studies. Specifically, the majority of this work has centered around the question of whether bottom-up salience can provide a robust explanation for the distribution of fixation locations during a variety of tasks such as free-viewing, visual search, and scene memorization. Furthermore, bottom-up salience is frequently taken as a benchmark to evaluate other factors against. For example, Tatler (2007) shows that there is a considerable bias toward fixating the center of an image; Einhauser et al. (2008) argue that people prefer to look at objects rather than low-level salient regions. Similarly, Nuthmann and Henderson (2010) argue that fixations are directed to the center of objects rather than salient regions; Torralba et al. (2006) show that a contextual map of where the target is likely to appear outperforms bottom-up salience in the prediction of fixation locations during visual search.

The work presented here shows that low-level visual salience plays an important role even in higher-level task-driven cognitive behavior. However, results like these suggest that a more object-centric model of visual attention might do even better. Our results support the idea of a close connection between vision and language, where relatively low-level mechanisms on one side can influence the other. We hope that further study of tasks like REG can reveal more about this interface and what kinds of information pass through it.

This study shows a clear effect of visual properties on the production of referring expressions, both in length and in composition. This conclusion may seem obvious – surely the complexity of the image people are looking at should affect what they say. But nonetheless, over a decade of research has failed to meaningfully establish it, producing instead a confusing array of weak results and failures to find significance. Moreover, this gap in the research record has had significant influence on the models proposed for REG. Psychological models like Gatt et al. (2012) propose a relatively limited role for vision in REG, which they treat as a pre-process reducing a visual scene to an unordered list of objects and assigning each one a set of categorical features. Computational models of REG similarly pay little attention to the perceptual underpinnings of vision – neither minimalist nor incremental models have gone beyond Kelleher et al.'s (2005) simplistic use of area as a proxy for visual salience. Without a clear demonstration of what kind of images are necessary to produce salience and clutter effects and how influential they can be, there is no motivation to incorporate such features into these models.

This paper should serve as to correct such views. For sufficiently complex images, visual features do matter, and the coefficients in our models make explicit predictions about *how much* a particular degree of corpus-wide variation in visual salience is expected to influence the results. In order to generalize to the full range of human performance, we argue that future models of REG should incorporate up-to-date models of low-level perception from the vision literature. Their performance should be evaluated on complex images with hundreds of objects, each differing in salience, as well as the arrays of ten or twenty similar-looking objects used in previous work. Finally, vision scientists working on salience should consider their models to be more than simple fixation predictors; visual salience has high-level cognitive effects which surface even in simple experiments.

### Conflict of interest statement

The authors declare that the research was conducted in the absence of any commercial or financial relationships that could be construed as a potential conflict of interest.

## Footnotes

^1^If it was unclear which object was the main object of the description and which the landmark, we preferred to make the target the main object. Then any landmarks that were directly relative to the target, and so on. This is because the participant's task was to refer to the target; other objects were presumably introduced to help locate it and not vice versa.

^2^We also investigated *log* (l + area), but the linear correlation with landmark choice is not as strong.

^3^Because this analysis relies only on the total number of words, regardless of what they describe, we perform the analysis on all 28 scenes. On the 11 annotated images, we obtain a similar result (*p* = 0.47) but with less power (*p* = 0.14).

^4^Although the number of words devoted to targets decreases as a proportion of the total as expressions lengthen, the absolute number of words devoted to targets increases.
