# Corrigendum: Where's Wally: the influence of visual salience on referring expression generation

**DOI:** 10.3389/fpsyg.2024.1445052

**Published:** 2024-07-26

**Authors:** Alasdair D. F. Clarke, Micha Elsner, Hannah Rohde

**Affiliations:** ^1^School of Informatics, University of Edinburgh, Edinburgh, Scotland, UK; ^2^Department of Linguistics, The Ohio State University, Columbus, OH, USA; ^3^Linguistics and English Language, University of Edinburgh, Edinburgh, Scotland, UK

**Keywords:** referring expression generation, visual salience, visual clutter

In the published article, there was an error in Figures 3, 4 as published. The license to use the Where's Wally image has expired. In the updated article, these figures have been removed. The remaining figures have been renumbered accordingly, and the text has been amended as outlined below to remove the previous figure citations.

A correction has been made to **Visual Search and Visual Salience**, Paragraph 4. Instead of “But we would argue that the real world looks more like Figure 3 than Figure 2.” The corrected sentence should be “But we would argue that the real world looks more complex than Figure 2.”

Corrections have also been made to **Materials and Methods**. Firstly, in “*Data Collection*,” Paragraph 1, instead of “A collection of 28 images taken from the *Where's Wally* picture books (Handford, 1987, 1988, 1993) were used as stimuli (see Figures 3 and 4).” The corrected sentence should be “A collection of 28 images taken from the *Where's Wally* picture books (Handford, 1987, 1988, 1993) were used as stimuli.”

In **Materials and Methods**, “*Annotation*,” Paragraph 1, instead of “Sample annotations are shown beneath the images in Figures 3 and 4.” The corrected wording should be “Sample annotations are shown in examples (1) and (2). Words in < TARG> tags describe the target. Example (1) shows the annotated referring expression for an easy stimulus; a single landmark (the burning hut, indicated by the REL attribute) is used to localize the target. Example (2) shows the expression for a harder stimulus; two landmarks (the umbrella and ball) are introduced with the word “find” and marked with < EST> tags, and the ball is then used to localize the target.” Therefore, the full corrected Paragraph 1 should be written as shown below.

“We annotated the elicited referring expressions to indicate which objects in the image were mentioned, which words in each expression referred to each object, and how the object references related to one another. Sample annotations are shown in examples (1) and (2). Words in < TARG> tags describe the target. Example (1) shows the annotated referring expression for an easy stimulus; a single landmark (the burning hut, indicated by the REL attribute) is used to localize the target. Example (2) shows the expression for a harder stimulus; two landmarks (the umbrella and ball) are introduced with the word “find” and marked with < EST> tags, and the ball is then used to localize the target. Objects in the image were labeled with bounding boxes (or for very large non-rectangular objects, bounding polygons). We did not distinguish references to geometrical parts of an object (“the left side of the track”) from references to the whole object, nor did we create separate boxes for small items that people wear or carry, or for architectural details of buildings (so “the boy in the yellow shirt” is treated as a single object). A few bounding boxes indicate groups of objects mentioned as a unit (“the three men”).”

This corrected paragraph refers to new examples (1) and (2), shown below, which are now inserted following Paragraph 1. (1) The < TARG> man < /TARG> just to the left of the < LMARK REL="TARG" OBJ="IMGID"> burning hut < /LMARK> < TARG> holding a torch and a sword < /TARG>. (2) Find < EST OBJ="IMGID1"> the red and white umbrella < /EST>. Then find < EST OBJ="IMGID2"> the blue and white beach ball < /EST>. Below and to the left < LMARK OBJ="IMGID2" REL="TARG"/> is < TARG> a dark skinned woman with a red bathing suit < /TARG>.

A correction has been made to **Materials and Methods**, “*Annotation*,” Paragraph 2. Instead of “so in Figure 4, “below and to the left” is annotated like “below and to the left *of the ball*”).” The corrected sentence should be “so in example (2), “below and to the left” is annotated like “below and to the left *of the ball*”).”

Lastly, in the published article, a correction has been made to **Discussion**, Paragraph 1. Instead of, “The beach scene (Figure 4), for instance, has hundreds of similarly sized and colored human figures which are generally poor choices as landmarks, since most of them are no easier to find than the targets.”, the corrected sentence appears below.

“The beach scene, for instance, has hundreds of similarly sized and colored human figures which are generally poor choices as landmarks, since most of them are no easier to find than the targets.”

The authors apologize for these errors and state that they do not change the scientific conclusions of the article in any way. The original article has been updated.
